# Cell-Permeable Succinate Rescues Mitochondrial Respiration in Cellular Models of Amiodarone Toxicity

**DOI:** 10.3390/ijms222111786

**Published:** 2021-10-29

**Authors:** Alina M. Bețiu, Imen Chamkha, Ellen Gustafsson, Elna Meijer, Vlad F. Avram, Eleonor Åsander Frostner, Johannes K. Ehinger, Lucian Petrescu, Danina M. Muntean, Eskil Elmér

**Affiliations:** 1 Department of Functional Sciences-Pathophysiology, “Victor Babeș” University of Medicine and Pharmacy, Eftimie Murgu Sq. no. 2, 300041 Timișoara, Romania; betiu.alina@umft.ro; 2 Centre for Translational Research and Systems Medicine, “Victor Babeș” University of Medicine and Pharmacy, Eftimie Murgu Sq. no. 2, 300041 Timișoara, Romania; avram.vlad@umft.ro; 3 Mitochondrial Medicine, Department of Clinical Sciences Lund, Faculty of Medicine, Lund University, BMC A13, 221 84 Lund, Sweden; imen.chamkha@med.lu.se (I.C.); ellen.gustafsson.6014@student.lu.se (E.G.); elna.meijer.1823@student.lu.se (E.M.); eleonor.asander_frostner@med.lu.se (E.Å.F.); johannes.ehinger@med.lu.se (J.K.E.); 4 Abliva AB, Medicon Village, 223 81 Lund, Sweden; 5 Department of Internal Medicine-Diabetes, Nutrition, Metabolic Diseases and Rheumatology, “Victor Babeș” University of Medicine and Pharmacy, Eftimie Murgu Sq. no. 2, 300041 Timișoara, Romania; 6 Institute of Cardiovascular Diseases, Doctoral School of “Victor Babeș” University of Medicine and Pharmacy, Eftimie Murgu Sq. no. 2, 300041 Timișoara, Romania; petrescu_lucian@yahoo.com

**Keywords:** amiodarone, desethylamiodarone, sotalol, NV118, platelets, PBMCs, HepG2 cells, mitochondria, respiration, ATP

## Abstract

Amiodarone is a potent antiarrhythmic drug and displays substantial liver toxicity in humans. It has previously been demonstrated that amiodarone and its metabolite (desethylamiodarone, DEA) can inhibit mitochondrial function, particularly complexes I (CI) and II (CII) of the electron transport system in various animal tissues and cell types. The present study, performed in human peripheral blood cells, and one liver-derived human cell line, is primarily aimed at assessing the concentration-dependent effects of these drugs on mitochondrial function (respiration and cellular ATP levels). Furthermore, we explore the efficacy of a novel cell-permeable succinate prodrug in alleviating the drug-induced acute mitochondrial dysfunction. Amiodarone and DEA elicit a concentration-dependent impairment of mitochondrial respiration in both intact and permeabilized platelets via the inhibition of both CI- and CII-supported respiration. The inhibitory effect seen in human platelets is also confirmed in mononuclear cells (PBMCs) and HepG2 cells. Additionally, amiodarone elicits a severe concentration-dependent ATP depletion in PBMCs, which cannot be explained solely by mitochondrial inhibition. The succinate prodrug NV118 alleviates the respiratory deficit in platelets and HepG2 cells acutely exposed to amiodarone. In conclusion, amiodarone severely inhibits metabolism in primary human mitochondria, which can be counteracted by increasing mitochondrial function using intracellular delivery of succinate.

## 1. Introduction

Cardiac arrhythmias are conditions defined by an irregular heartbeat that can arise from either atria or ventricles [1]. Amiodarone is the most effective class III (Vaughan-Williams’ classification) antiarrhythmic drug, widely used to treat both ventricular and supraventricular arrhythmias [2]. The new European Society of Cardiology Guidelines for the diagnosis and management of atrial fibrillation recommend amiodarone for long-term rhythm control in all patients with atrial fibrillation, including those with heart failure [3]. Desethylamiodarone, the main metabolite of amiodarone, also possesses antiarrhythmic properties via the N-demethylation reaction catalyzed by cytochrome P450 3A4 [4,5]. Amiodarone and DEA quickly and extensively accumulate in extracardiac tissues (mainly in the adipose tissue) after amiodarone administration, reaching concentrations up to 1000 times higher than the equivalent plasma concentrations, and have a long elimination half-life [6,7,8]. Moreover, amiodarone and its metabolite DEA can cause several extracardiac side effects, mainly hepatic and pulmonary toxicity, but also thyroid dysfunction, which may lead to treatment discontinuation [5,9,10,11].

Despite extensive research, the exact mechanism responsible for human amiodarone toxicity is only partially elucidated, but evidence indicates mitochondrial dysfunction and oxidative stress as two key factors in amiodarone toxicity [12,13]. In mammalian cells amiodarone has been shown to impair mitochondrial respiration, mainly the function of mitochondrial complexes I (CI) and II (CII) of the electron transport system (ETS) [14,15,16]. The group of Gallyas Jr. first reported the concentration-dependent effects of amiodarone in isolated cardiac and liver rat mitochondria, showing no effect at low concentrations (up to 6 µM), uncoupling between 6–30 µM and inhibition of the respiratory chain at higher concentrations, respectively [17]. Recently, cell-permeable succinate prodrugs have been developed in order to bypass the mitochondrial complex I deficiency of different etiologies [18,19,20]. These prodrugs enter the cells independent of active uptake and, upon cleavage by intracellular esterases, subsequently release succinate, the complex II substrate. In addition to bypassing the complex I dysfunction, the prodrugs have been reported to alleviate metabolic dysfunction by increasing electron transport in situations of partial inhibition, and also in downstream complexes [21].

Sotalol, a non-cardioselective beta-blocker classified as a class III antiarrhythmic agent due to its predominant potassium channel blocking effect, could be used as an alternative to amiodarone, if there are no contraindications, having a more favorable adverse-event profile [22].

Blood cells, and especially platelets, have recently been recognized as convenient tools to assess mitochondrial respiration, mirroring organ-specific mitochondrial dysfunction in various pathologies [23]. Platelets serve as an easily obtainable source of human viable mitochondria when studying mitochondrial toxic effects elicited by different drug exposure [24].

The objectives of the present study were: (i) to assess the effects of amiodarone, DEA and sotalol (as a negative control of mitochondrial toxicity) on mitochondrial respiration in human platelets, (ii) to evaluate the efficacy of a cell-permeable succinate prodrug (diacetoetoxymethyl succinate, NV118) in alleviating the acute drug-induced mitochondrial dysfunction, (iii) to probe whether the effect on respiration could be confirmed in more complex cells, e.g., peripheral blood mononuclear cells (PBMCs) and HepG2 cells, and also, to assess if the effect on respiration would translate into an inhibition of cellular ATP synthesis.

## 2. Results

### 2.1. Concentration-Dependent Decrease of Mitochondrial Respiration by Amiodarone, but Not Sotalol in Intact Human Platelets

**Figure 1:**
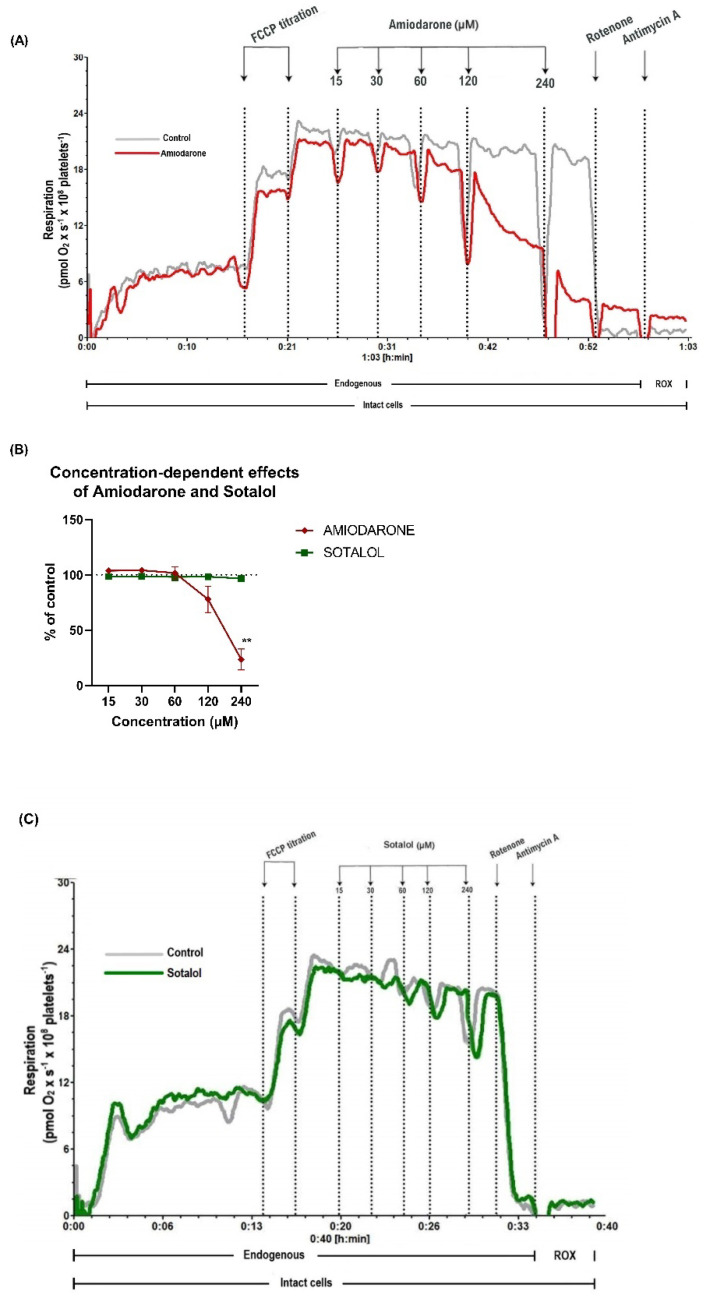
Amiodarone, but not sotalol, induced concentration-dependent impairment of mitochondrial respiration in intact platelets. (**A**) Representative trace of amiodarone-induced mitochondrial dysfunction (amiodarone-red; DMSO-grey). (**B**) Respiration of human platelets was measured after submaximal uncoupling with FCCP (2 µM). The concentration-dependent effect of amiodarone (red rhombus) and sotalol (green square) was measured by titrating increasing concentrations of each drug (15–240 µM) vs. the equivalent volume of vehicle (DMSO or H_2_O). Non-mitochondrial respiration was evaluated by the inhibition of complex I with rotenone (2 µM) and complex III with antimycin A (1 µg/mL). (**C**) Representative trace of sotalol effect on mitochondrial respiration (sotalol-green; H_2_O/solvent-grey). Data were expressed as mean ± SEM. Two-way ANOVA with Bonferroni post hoc test was performed on the antimycin-corrected data. ROX: residual oxygen consumption. DMSO: dimethyl sulfoxide. FCCP: carbonyl cyanide p-(trifluoromethoxy) phenylhydrazone. ** *p* < 0.01 vs. DMSO. *n* = 5.

Mitochondrial respiration of intact human platelets was assessed in the presence of increasing concentrations of amiodarone and sotalol (15–240 µM), respectively. Significant concentration-dependent respiratory inhibition was elicited by amiodarone with a reduction of mitochondrial oxygen consumption to 23.4% ± 9.5 (*p* < 0.01) of control (Figure 1A,B) at the highest concentration, whereas sotalol did not elicit toxic effects even at 240 µM (Figure 1B,C).

### 2.2. Amiodarone, but Not Sotalol, Elicited a Concentration-Dependent Decrease of Mitochondrial Respiration in Permeabilized Human Platelets

**Figure 2:**
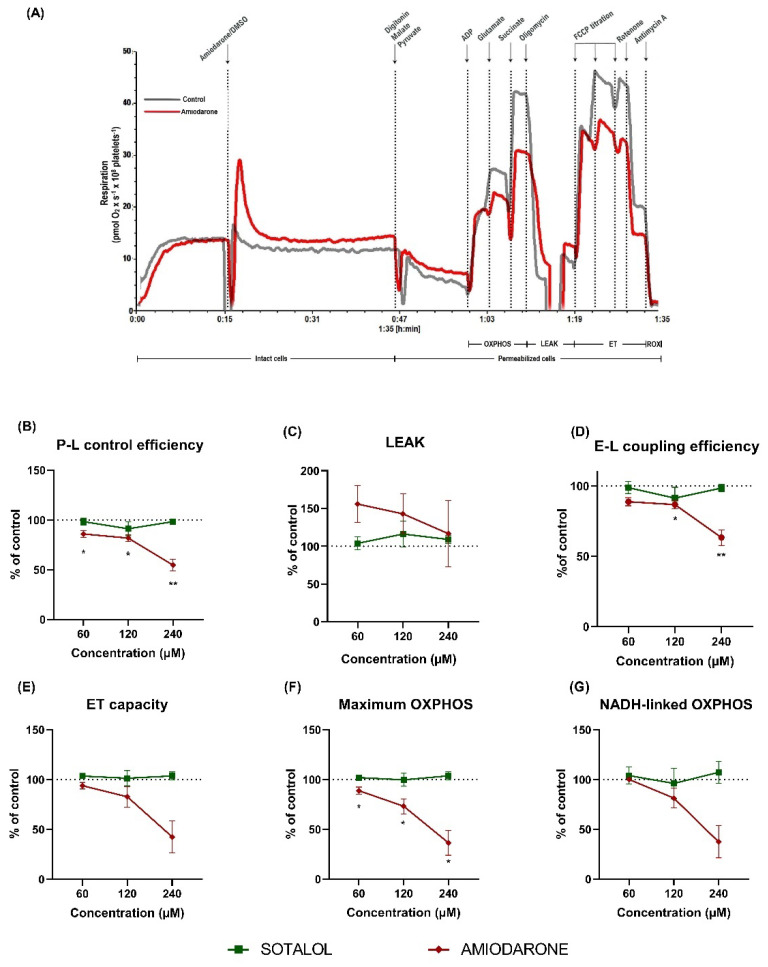

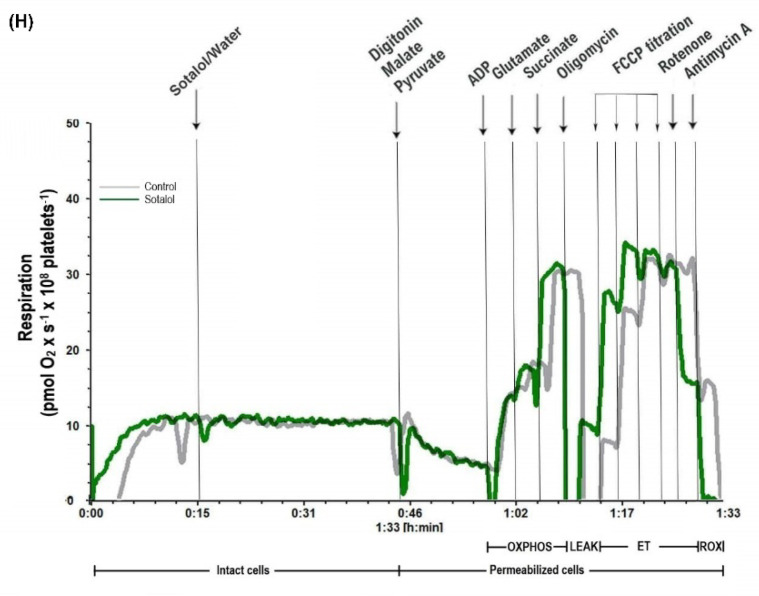
Amiodarone (but not sotalol) induced a concentration-dependent inhibition of mitochondrial respiration in permeabilized platelets. (**A**) Representative trace of amiodarone-induced mitochondrial dysfunction (amiodarone-red; DMSO/solvent-grey). Concentration-dependent effects were assessed for 3 doses (60 µM, 120 µM and 240 µM) of amiodarone (red rhombus) and sotalol (green square), respectively. P-L control efficiency, a measure of ATP generating respiration (**B**), LEAK (**C**), E-L coupling efficiency, a measure of ATP generating respiration (**D**), ET capacity (**E**), maximum OXPHOS (**F**), NADH-linked OXPHOS (**G**) were evaluated. (**H**) Representative trace of sotalol effects (sotalol-green; H_2_O/solvent-grey). Data were expressed as mean ± SEM of the percent of control (platelets exposed to the corresponding volume of DMSO and H_2_O for highest concentration of drug). Two-way ANOVA with Bonferroni post hoc test was performed on the antimycin-corrected data. DMSO: dimethyl sulfoxide. ADP: adenosine diphosphate. FCCP: carbonyl cyanide p-(trifluoromethoxy) phenylhydrazone. OXPHOS: oxidative phosphorylation. LEAK: non-phosphorylating respiration. ET: electron transfer. ROX: residual oxygen consumption. * *p* < 0.05, ** *p* < 0.01 vs. control. *n* = 5.

In order to further characterize the mechanisms of the concentration-dependent electron transfer system (ETS) impairment elicited by the antiarrhythmic drugs, mitochondrial respiration was evaluated in permeabilized platelets using three different concentrations (60, 120 and 240 µM) of amiodarone and sotalol, respectively (representative traces of amiodarone and sotalol effects are shown in Figure 2A,H).

P-L control efficiency is a measure of ATP-generating mitochondrial oxygen consumption and was calculated according to [25] as 1-LEAK/maximum OXPHOS. Amiodarone lowered P-L control efficiency (Figure 2B) already at 60 µM, reaching 85.9% ± 3.3 of control (*p* < 0.05) with a further decrease to 54.7% ± 5.9 of control (*p* < 0.01) in the presence of the highest concentration (240 µM).

LEAK respiration (non-phosphorylating respiration) demonstrated a trend towards increasing in the presence of amiodarone (Figure 2C), with the highest increase at 60 µM (155.9% ± 24.2 of control) and the mildest at 240 µM (116.7% ± 43.8), but with no statistical significance.

E-L coupling efficiency is another measure used to assess the efficiency of ATP generation and was calculated according to [25] as 1-LEAK/ET capacity. It was impaired by amiodarone (Figure 2D) reaching 86.7% ± 2.9 of control at 120 µM (*p* < 0.05) with a further decrease to 63.3% ± 5.5 of control (*p* < 0.01) at 240 µM.

ET capacity, which mirrors the maximal activity of the ETS (Figure 2E), was also affected in a similar fashion, reaching 42.6% ± 16 of control at 240 µM; however, there was no statistical significance.

Maximum OXPHOS, corresponding to the sum of NADH-linked OXPHOS and succinate-linked OXPHOS, was decreased by amiodarone in a concentration-dependent manner (Figure 2F) reaching 36.4% ± 12.5 of control (*p* < 0.05) at 240 µM. When separately assessing the OXPHOS pathways, NADH-linked OXPHOS (Figure 2G) showed a comparable decrease (37.4 ± 16.1 of control at 240 µM amiodarone).

### 2.3. Characterization of DEA-Induced Mitochondrial Dysfunction in Permeabilized Human Platelets

**Figure 3:**
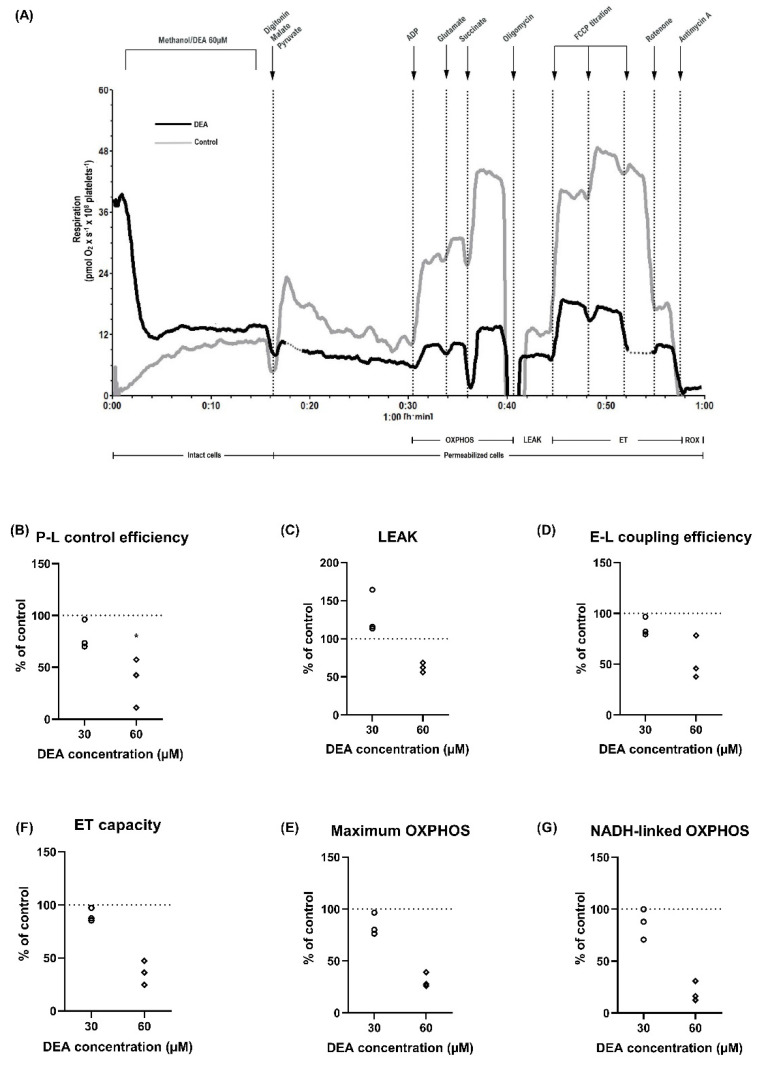
The amiodarone metabolite (DEA) induced a concentration-dependent inhibition of mitochondrial respiration in permeabilized platelets. (**A**) Representative trace of DEA exposure (DEA-black; methanol-grey). Concentration-effects for two concentrations of DEA (30 µM-circle and 60 µM-rhombus). P-L control efficiency, a measure of ATP generating respiration (**B**), LEAK (**C**), E-L coupling efficiency, a measure of ATP generating respiration (**D**), ET capacity (**E**), maximum OXPHOS (**F**), NADH-linked OXPHOS (**G**) were evaluated. Data were expressed as individual values of the percent of control (platelets exposed to the corresponding volume of methanol). Two-way ANOVA with Bonferroni post hoc test was performed on the antimycin-corrected data. DMSO: dimethyl sulfoxide. ADP: adenosine diphosphate. FCCP: carbonyl cyanide p-(trifluoromethoxy) phenylhydrazone. OXPHOS: oxidative phosphorylation. LEAK: non-phosphorylating respiration. ET: electron transfer. ROX: residual oxygen consumption. * *p* < 0.05 vs. control. *n* = 3.

Amiodarone may trigger mitochondrial dysfunction not only in a direct manner, but also through the accumulation of its metabolite DEA. The effects of DEA on mitochondrial oxygen consumption were evaluated by assessing the same parameters of high-resolution respirometry as for amiodarone (a typical trace of DEA effects is presented in Figure 3A).

DEA elicited a concentration-dependent reduction of P-L control efficiency (Figure 3B) to 37% ± 13.7 of control (*p* < 0.05) at 60 µM. At the highest concentration used (60 µM) all the examined parameters were reduced compared to the control as follows: LEAK respiration (Figure 3C) to 62.2% ± 3.6, E-L coupling efficiency (Figure 3D) to 53.9% ± 5.4, ET capacity (Figure 3E) to 36.1% ± 6.5, maximum OXPHOS (Figure 3F) to 31% ± 4.1, NADH-linked OXPHOS (Figure 3G) to 20% ± 5.6; however, no statistical significance was reached.

### 2.4. Amiodarone Elicited a Time- and Concentration-Dependent Decrease in Mitochondrial Respiration Combined with ATP Depletion in Intact PBMCs

In order to determine whether the results obtained in platelets exposed to amiodarone can be recapitulated in other metabolically more active blood cells, mitochondrial oxygen consumption was further evaluated in PBMCs. Since amiodarone elicited deleterious effects on both maximum OXPHOS (Figure 2F) and NADH-linked (Figure 2G) OXPHOS, we assessed the effects of amiodarone and rotenone (as the positive control for complex I-inhibition) on mitochondrial respiration and cellular ATP levels.

**Figure 4:**
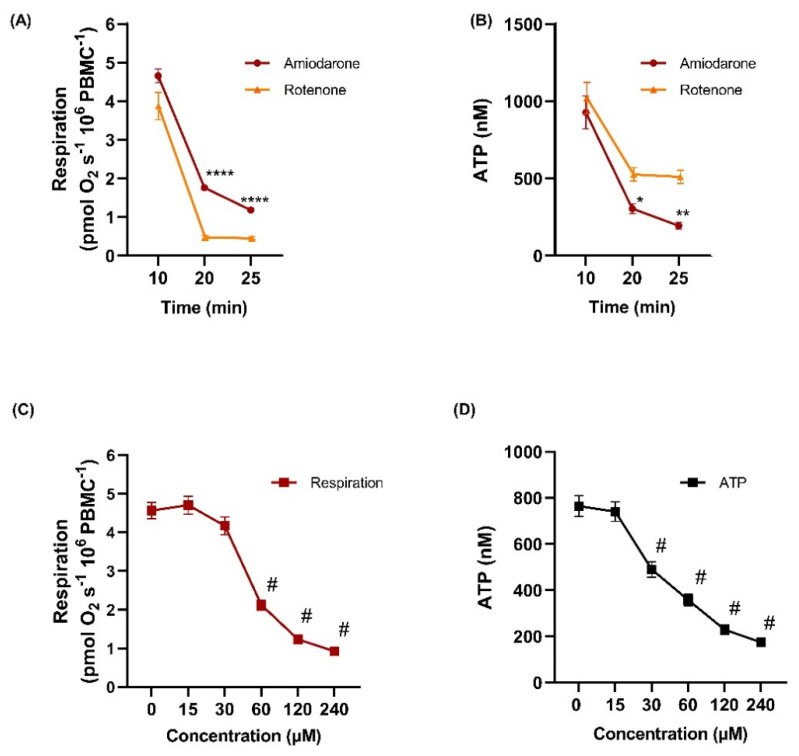
Delayed toxicity and ATP-depletion by amiodarone in mononuclear cells. Temporal decline of respiration (**A**) and ATP levels (**B**) was observed in intact PBMCs following single administration of amiodarone (100 µM, red) and rotenone (2 µM, orange), added on routine (basal) respiration. (**C**) Oxygen consumption (red square) and (**D**) ATP concentration (black square) followed amiodarone titration. Amiodarone was added at 10, 15, 20, 25 and 30 min at the final concentrations of 15, 30, 60, 120 and 240 μM. Data were expressed as mean ± SEM. Two-way ANOVA with Bonferroni post hoc test was performed to evaluate time-dependent effects of amiodarone and rotenone (**A**,**B**) panels, * *p* < 0.05; ** *p* < 0.01; **** *p* < 0.0001 vs. rotenone. One-way ANOVA with Bonferroni post hoc test was performed to evaluate dose-dependent effects between amiodarone and rotenone (**C**,**D**) panels, # *p* < 0.0001 vs. 0 µM. *n* = 5–6.

A single addition of amiodarone (100 µM) and rotenone (2 µM), respectively, caused a time-dependent decrease in mitochondrial oxygen consumption (pmoL O_2_ s^−1^ 10^6^ cells^−1^) from 4.7 ± 0.18 at 10 min to 1.2 ± 0.03 at 25 min and 3.8 ± 0.35 at 10 min to 0.4 ± 0.04 at 25 min, respectively (Figure 4A). A time-dependent decrease in the ATP content (Figure 4B) was also seen after exposure to amiodarone (100 µM), from 928.7 ± 106.58 nM at 10 min to 194.3 ± 22.19 nM at 25 min. Rotenone (2 µM) caused a decrease from 956.52 ± 112.85 nM to 510.3 ± 42.69 nM for the same time points. When compared at 25 min of exposure, amiodarone elicited a significant inhibition of respiration in PBMCs but its effect did not surpass the inhibition elicited by rotenone (*p* < 0.0001) whereas the decrease in the ATP levels induced by amiodarone surpassed the one elicited by rotenone, which implies additional toxicity besides the mitochondrial toxicity (*p* < 0.01).

The effects on mitochondrial respiration and ATP levels with increasing concentrations up to 240 µM of amiodarone were evaluated in intact PBMCs (Figure 4C,D). Significant respiratory inhibition was elicited by amiodarone starting from 60 µM (*p* < 0.0001), while the drop in ATP level was significant already at 30 µM (*p* < 0.0001). As evident from Figure 4A, there is a significant time-lag of the amiodarone-induced inhibition of respiration and ATP production, hence the inhibition curves of Figure 4C are underestimated.

#### 2.4.1. Treatment Effect of a Cell-Permeable Succinate Prodrug on Amiodarone-Induced Mitochondrial Dysfunction in Human Platelets

Amiodarone caused mitochondrial dysfunction via the inhibition of both NADH and succinate-linked respiration, with an increased toxicity observed in case of the latter (Figure 2). Subsequently, we investigated to what degree this dual respiratory impairment can be alleviated by the cell-permeable succinate (NV118) in platelets acutely exposed to 120 µM amiodarone.

**Figure 5:**
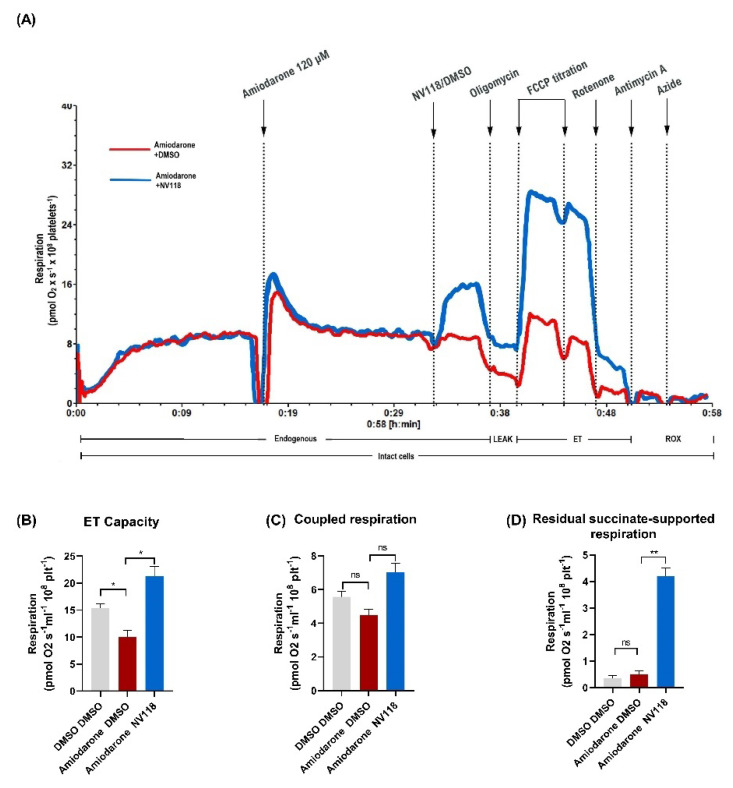
Effects of the succinate prodrug NV118 on amiodarone-induced mitochondrial dysfunction in human platelets**.** (**A**) Representative trace of amiodarone-exposed platelets in the absence (red) or presence (blue) of NV118. (**B**–**D**) NV118 effects on amiodarone-exposed platelets (blue) were measured as compared to its vehicle (DMSO, red). As negative control platelets were exposed to only DMSO (grey). Data were expressed as mean ± SEM. One-way ANOVA with Bonferroni post hoc test was performed on the antimycin-corrected data. DMSO: dimethyl sulfoxide. FCCP: carbonyl cyanide *p*-(trifluoromethoxy) phenylhydrazone. LEAK: non-phosphorylating respiration. ET: electron transfer. ROX: residual oxygen consumption. ns = not significant. * *p* < 0.05; ** *p* < 0.01 vs. DMSO. *n* = 5.

A representative overlay trace of amiodarone-exposed platelets in the presence (blue) vs. absence (red) of NV118 is depicted in Figure 5A. The addition of the prodrug to the amiodarone-exposed platelets resulted in an increase in the ET capacity (Figure 5B) above the control levels (*p* < 0.05) and in levels of coupled respiration (ATP generating respiration) similar to those of the control samples (Figure 5C), respectively. These effects are due to an increase in succinate-supported respiration (Figure 5D), measured as oxygen consumption after the inhibition of complex I.

#### 2.4.2. Cell-Permeable Succinate-Alleviated Mitochondrial Dysfunction Induced by Amiodarone in HepG2 Cells

**Figure 6:**
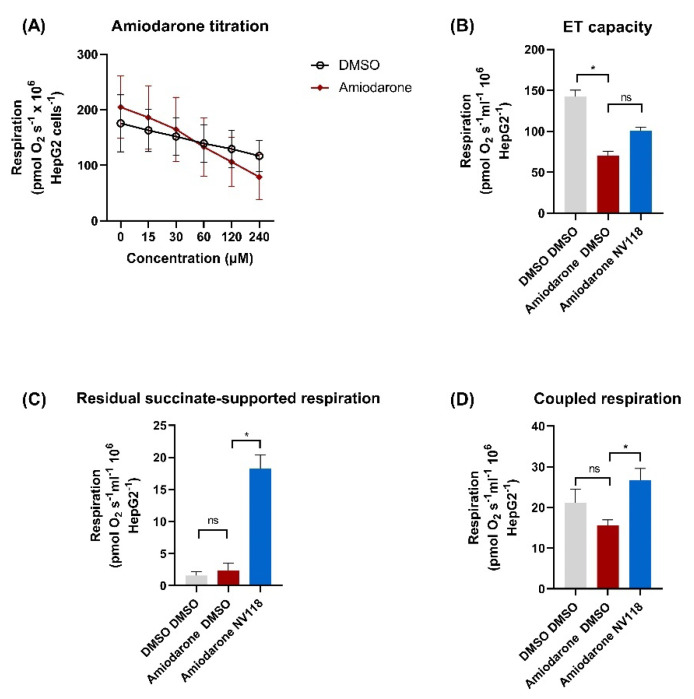
Effects of NV118 on amiodarone-induced mitochondrial dysfunction in human HepG2 cells. (**A**) Respiration of HepG2 cells was measured after mild uncoupling with FCCP (4 µM). The dose-dependent effect of amiodarone (red rhombus) was measured by titrating increasing concentrations of amiodarone or vehicle (DMSO, open black circle). Non-mitochondrial respiration was evaluated by the inhibition of complex I with rotenone (2 µM) and that of complex III with antimycin A (1 µg/mL). (**B**–**D**) NV118 effects (blue) on amiodarone-treated HepG2 cells were assessed as compared to its vehicle (DMSO, red). As negative control of the experiment HepG2 were exposed only to DMSO (grey). Data were expressed as mean ± SEM. Two-way ANOVA and one-way ANOVA with Bonferroni post hoc test were performed on the antimycin-corrected data. DMSO: dimethyl sulfoxide. ET: electron transfer. ns= not significant. * *p* < 0.05 vs. DMSO. *n* = 3–4.

Since amiodarone hepatotoxicity is a common side effect, we further evaluated whether the beneficial effects of NV118 can be recapitulated in a liver cell line (HepG2 cells). Oxygen consumption (pmoL O_2_ s^−1^ 10^6^ cells) of HepG2 cells was assessed after exposure to the same concentrations of amiodarone used in the previously described protocol in intact platelets. Mitochondrial respiration was reduced to 34% ± 8.4 of control at 240 µM (Figure 6A). The addition of NV118 to the amiodarone-exposed HepG2 cells elicited results comparable to those obtained in human platelets. Thus, ET capacity was 101 ± 7.4 in the amiodarone-treated cells receiving NV118 as compared to 70.6 ± 5.2 in the amiodarone-treated control cells (Figure 6B), and the effects were mediated by the increase of succinate-supported mitochondrial oxygen consumption (Figure 6C), measured as oxygen consumption after the inhibition of complex I. Comparable effects on coupled respiration were found as the NV118-treated samples had higher rates than the control samples (Figure 6D), with an oxygen consumption of 15.6 ± 1.4 in control samples and 26.7 ± 2.9 in the samples receiving NV118 (*p* < 0.05).

## 3. Discussion

In recent years, mitochondrial dysfunction has been increasingly recognized as a key pathomechanism underlying the adverse reactions of many drugs. The fact that mitochondria represent a target of drug toxicity is not surprising, since these organelles play a central role in energy production via multiple metabolic pathways and are key players in the coordination of several types of cell death [26].

Mitochondria-related toxicity was mainly demonstrated in the literature in murine models where amiodarone elicited uncoupling of oxidative phosphorylation, inhibition of the electron transfer system and also, inhibition of fatty acid ß-oxidation [16,27,28,29]. We have shown here that amiodarone causes a concentration-dependent reduction in mitochondrial oxygen consumption of human platelets, leading to an overall decrease in P-L control efficiency. As depicted in Figure 2C,F, in permeabilized platelets amiodarone elicited a mild increase in LEAK respiration (maximal at 60 μM and lower at higher concentrations) together with a significant, progressive decrease in OXPHOS (both CI- and CII- supported, starting from 60 μM). Our results are in line with early studies performed in murine-isolated liver mitochondria pointing to the dual, dose-dependent in vitro effect of amiodarone on mitochondrial respiration, which consisted in a transient (yet significant) uncoupling effect at low concentrations, while acting as a CI and CII inhibitor when applied in higher doses [16,29]. Regarding the ATP depletion, our data are in line with the results published by Felser et al., which showed in isolated rat mitochondria and human hepatocytes that in vitro exposure to amiodarone leads to a decrease in the intracellular ATP content [15]. Moreover, in a rat model of hepatotoxicity, amiodarone has been shown to increase liver mitochondrial hydrogen peroxide formation and induce cardiolipin peroxidation, accompanied by inhibition of mitochondrial complex I activity, uncoupling of oxidative phosphorylation and a decrease in liver ATP levels [30]. A reduction in the ATP levels is detrimental to cellular function [31] and explains why amiodarone may induce a concentration-dependent increase in cell death in HepG2 cells [32].

Amiodarone caused a potent inhibition of maximum OXPHOS. Previous studies described the role of oxaloacetate, whose production depends on complex I stimulation, as a potent inhibitor/modulator of complex II [33,34]. In the present experiments, due to the low activity of complex I in amiodarone-exposed cells, it is tempting to speculate that complex II is less subjected to oxaloacetate inhibition and thereby both complex I and complex II-supported OXPHOS were reduced. While in our hands amiodarone caused a more potent inhibition of complex II-supported respiration in human platelets, a study conducted in isolated rat heart mitochondria revealed that amiodarone inhibited both complex I and II of the ETS with a more potent inhibition of the NADH-dependent respiration, thus suggesting that amiodarone-induced mitochondrial dysfunction is species/tissue dependent [35]. Of note, these authors also reported that amiodarone acted as an uncoupler in the heart, as demonstrated here in human platelets.

We have further demonstrated that not only amiodarone, but also its metabolite (DEA) elicits platelet mitochondrial dysfunction. Takai et al. demonstrated in an in vivo mouse model that the detrimental effects on mitochondrial function and the degree of liver injury induced by amiodarone administration correlated with the plasma levels of DEA [36]. Bolt et al. reported that both amiodarone and DEA inhibited complex I- and complex II-supported respiration in hamster lung macrophages; interestingly, DEA inhibited complex II to a greater degree than did amiodarone in whole lung mitochondria [14].

At variance from platelets, which are corpuscular fragments originating from megakaryocytes [37], PBMCs are nucleated cells [38]. Regardless of this, amiodarone dose-dependently decreased mitochondrial respiration in both blood cell types, an observation important for the generalizability of our results. In PBMCs we have also demonstrated that amiodarone-depressed respiration was dose-dependently associated with a decreased ATP content. The fact that amiodarone lowered the ATP levels more than did rotenone (which fully inhibited mitochondrial respiration), strongly suggests that the toxic effects of the drug goes beyond mitochondrial toxicity, i.e., a concomitant impairment of glycolysis, the other main source of cellular ATP, may be occurring. Our results are in line with the study of Fromenty et al., which reported that amiodarone caused a dramatic decrease in cellular ATP and cell viability in human lymphocytes, with a rate of ATP depletion twice as high with amiodarone as compared to rotenone [39]. Similarly, Karkhanis et al. have demonstrated that amiodarone significantly lowered the ATP content in rat H9c2 cardiomyocytes [35]. In contrast to the results of Fromenty et al., which reported that glucose was able to prevent ATP depletion (and thus decrease the amiodarone-induced human lymphocyte cytotoxicity), the ATP decrease in PBMCs occurred regardless of the presence of glucose (5 mM) in the medium [39]. Also, Bolt et al. reported that the inability of glucose to prevent amiodarone-induced depletion of ATP correlated with its lack of effectiveness against lung cell cytotoxicity [14]. Serviddio et al. showed that amiodarone did not impair ATP synthase activity but decreased the ATP availability; the authors speculated that this occurred via an increase in LEAK respiration with a decrease in the electrochemical gradient, which in turn, decreased the proton flux through ATP synthase [30].

At therapeutic dosage, amiodarone reaches plasma concentrations in the range of ~2 μM [40]. However, the tissue concentrations of both amiodarone and its metabolite (DEA) are higher than plasma concentrations [7]. For example, in liver tissue, amiodarone concentrations are 10–20 times higher than in plasma [15]. Amiodarone is metabolized in liver and produces DEA via cytochrome p450 (CYP3A4) [41]. Induction of CYP3A4 is a risk factor for hepatotoxicity, because the N-dealkylated metabolites were reported to be more hepatotoxic than amiodarone [32,41]. Since we have demonstrated that amiodarone-induced mitochondrial dysfunction is concentration-dependent, we speculate that concomitant association between amiodarone and inducers of cytochrome p450, such as carbamazepine, phenobarbital, phenytoin, rifampicin, isoniazid, tobacco, St. John’s Wort, ritonavir, omeprazole, dexamethasone or chronic alcohol consumption, may lead to more potent inhibition [42,43,44,45].

With ageing, a reduction in the oxidative capacity and ATP generation per mitochondrial volume is supposed to occur together with an increase in reactive oxygen species (ROS) generation [46,47]. ROS themselves induce further mitochondrial damage that, in turn, may predispose to cardiac arrhythmias [46,47,48]. The incidence of atrial fibrillation (which is treated with amiodarone) increases with ageing [2,49], thus amiodarone will be used in patients with already impaired mitochondria due to advanced age.

It should be noted that amiodarone is an inhibitor of cytochrome p450 and as such increases the concentration of drugs metabolized via this pathway, such as statins. It is well documented that drug-drug interactions may be a cause of statin-induced rhabdomyolysis [50,51]. Indeed, mitochondrial dysfunction is central to the deleterious effects of statins on skeletal muscle cells [52,53], but possibly not in blood cells, as recently reported [54]. However, since statin-induced mitochondrial dysfunction is also concentration-dependent [20], amiodarone may cause further mitochondrial damage via additive or synergic inhibition of mitochondria by the two drugs.

Recently, cell permeable succinates have been used to bypass NADH-linked mitochondrial dysfunction [18,19,55]. We have showed here that NV118 improved mitochondrial oxygen consumption in both platelets and HepG2 cells exposed to high concentrations of amiodarone. Complex II uses succinate as a substrate in an oxidation reaction that allows the electron transfer across the ETS, leading to proton translocation and the establishment of a proton gradient across the inner membrane of the mitochondria [19,25]. This gradient is essential for ATP generation [25]. NV118 delivers succinate to the mitochondria, providing increased substrate supply to complex II [18] and hence, an improved electron transport and proton gradient. Here we demonstrated that a cell-permeable succinate could increase metabolism through complex II despite a potential decrease in CII function by amiodarone. We conclude that amiodarone severely inhibits metabolism in primary human mitochondria, which can be counteracted by increasing mitochondrial function using intracellular delivery of succinate.

## 4. Materials and Methods

### 4.1. Chemicals and Human Samples

All chemicals were purchased from Sigma-Aldrich (Saint Louis, MO, USA). NV118 (cell-permeable succinate prodrug) was generously donated by Abliva AB (Lund, Sweden). This compound is also available as the MitoKit-CII from Oroboros Instruments (https://www.oroboros.at/index.php/product/mitokit-cii/) (accessed on 1 September 2021).

Human blood cells (platelets and PBMCs) isolation was carried out from venous blood samples provided by a group of healthy volunteers, both men and women. The blood was drawn in K_2_EDTA tubes. Isolation was achieved by means of differential centrifugation, as previously described by Sjövall et al. [24]. Oxygen consumption was normalized to the number of cells.

### 4.2. Cell Lines and Cell Culture

The human hepatocyte carcinoma cell line HepG2 (male, Caucasian, 15 years of age) was purchased from Sigma-Aldrich Chemie GmbH (Schnelldorf, Germany).

### 4.3. High-Resolution Respirometry (HRR)

HRR measurements were performed using the O2k-Oxygraph (Oroboros Instruments GmbH, Innsbruck, Austria) and two buffers: the MiR05 buffer containing: 0.5 mM EGTA, 3 mM MgCl_2_, 60 mM K-lactobionate, 20 mM taurine, 10 mM KH_2_PO_4_, 20 mM HEPES, 110 mM sucrose and 1 g/L bovine serum albumin [56] for platelets and HepG2 experiments and PBS buffer containing 137 mM NaCl, 2.7 mM KCl, 10 mM Na_2_HPO_4,_ 1.8 mM KH_2_PO_4_ with 5 mM glucose for PBMCs experiments. Respirometry protocols were performed at 37 °C, with 2 mL chamber volume and 750 rpm stirrer speed using cell concentrations of 200 × 10^6^ platelets/mL, 10^6^ PBMCs/mL and 0.5 × 10^6^ HepG2 cells/mL. The acute effects of the drug and metabolite exposure on mitochondrial respiration were evaluated by means of 4 different protocols:

Protocol I-Intact human platelets. Intact human platelets were subjected to FCCP-induced submaximal uncoupling (2 µM), to increase the resolution of the potential negative effects of amiodarone and sotalol on mitochondrial oxygen consumption, as compared to the corresponding volume of the control (DMSO, water). Increasing concentrations of amiodarone/sotalol were then titrated into the chamber (from 15 to 240 µM). In order to assess the contribution of non-mitochondrial respiration, complex I inhibition was obtained using rotenone (2 µM) and complex III inhibition with antimycin A (1 µg/mL). In order to determine the generalizability of these results in other cells, the same experiments with amiodarone were recapitulated in the HepG2 cell line.

Protocol II-Permeabilized human platelets. Mitochondrial respiration in permeabilized human platelets was measured in the presence of three different concentrations of amiodarone and sotalol (60, 120 and 240 µM) and compared to DMSO or water respectively (volume corresponding to the one used at the highest concentration of amiodarone/sotalol). The concentrations used in this set of experiments were determined in a series of pilot experiments (data not shown). Following the exposure to the drug or solvent, plasma membrane was permeabilized using a mild detergent, digitonin (1 µg/L × 10^6^ platelets) in order to allow the access of substrates. Malate (5 mM), pyruvate (5 mM), ADP (1 mM) and glutamate (5 mM) were added in order to activate complex I-supported respiration, after which succinate (10 mM) was added to further induce complex II-supported respiration. Subsequently, ATP-synthase was inhibited by oligomycin (1 μg/mL) to evaluate LEAK respiration (non-ATP generating respiration). The noncoupled maximal ETS capacity was determined by titrating FCCP up to complete dissipation of the proton gradient. The respirometric protocol was completed by measuring non-mitochondrial respiration following the addition of complex I inhibitor, rotenone (2 µM) and the complex III inhibitor, antimycin A (1 µg/mL), respectively [24]. For DEA the chosen concentrations were 30 and 60 µM and compared to vehicle (methanol). The concentrations were chosen after running a series of pilot experiments to identify the doses affecting mitochondrial function (data not shown).

Protocol III-Intact human platelets and prodrug treatment. Intact platelet respiration was measured in the presence of an amiodarone concentration of 120 µM (that elicited a decrease in mitochondrial respiration in previous experiments). NV118 (500 µM) was then added in the attempt to bypass mitochondrial complex I dysfunction (vs. DMSO). Coupled respiration was calculated as the difference before and after the addition of the ATP-synthase inhibitor, oligomycin (1 μg/mL). To achieve maximal ETS activity, consecutive titrations of FCCP were added, thus inducing a maximal noncoupled state. To evaluate the possibility of non-mitochondrial respiration elicited by the prodrug, complex I was inhibited with rotenone (2 µM), complex III with antimycin A (1 µg/mL), and complex IV with sodium azide (10 mM). As a control, mitochondrial oxygen consumption in platelets exposed to the volume of DMSO corresponding to the injection volume of amiodarone and that of the prodrug, was also measured.

In order to determine the generalizability of these results in other cells, the same experiments with amiodarone were recapitulated in the HepG2 cell line. Intact HepG2 cells respiration was measured in the presence of an amiodarone concentration of 60 µM (which elicited a decrease in mitochondrial respiration in previous experiments).

Protocol IV-Intact PBMCs. Intact PBMCs respiration and ATP levels were measured in the presence of an amiodarone concentration of 100 µM and 2 μM rotenone, respectively. Amiodarone (100 µM) or rotenone (2 µM) was added at 10, 15, 20, 25 and 30 min on ROUTINE (basal) respiration. In a separate experiment, amiodarone was titrated (15–240 μM) on routine (basal) respiration. After the highest dose of amiodarone (240 μM) was given, complex I linked respiration was inhibited by rotenone (2 µM). Non-mitochondrial respiration was measured by the addition of antimycin A (1 µg/mL). ATP samples were collected prior to amiodarone/rotenone addition and at determined time points thereafter. Respiration data were matched to the time points of ATP sample collection. Samples of chamber cell suspension were lysed with 2/3 ATPlite lysis solution and kept in −80 °C prior to ATP quantification.

Respiratory parameters-definitions and calculation (as previously presented in [20,57]):

- ROUTINE respiration: mitochondrial oxygen consumption in the physiological coupling state;
- LEAK respiration (non-phosphorylating respiration): mitochondrial oxygen consumption after inhibition of ATP-synthase;
- ET capacity: mitochondrial oxygen consumption in a fully uncoupled state achieved by the titration of optimum concentration of FCCP (protonophore);
- NADH-linked OXPHOS capacity: mitochondrial oxygen consumption at saturating concentrations of ADP and complex I substrates;
- OXPHOS capacity (phosphorylating respiration): mitochondrial oxygen consumption at saturating concentrations of ADP with both complex I and complex II substrates;
- Residual succinate-supported respiration: mitochondrial oxygen consumption after inhibition of complex I using rotenone;
- P-L control efficiency: calculated by subtracting LEAK respiration from OXPHOS capacity and then dividing the result by the OXPHOS capacity, it is a measure of the state of coupling (ATP generation) of the ETS;
- E-L coupling efficiency: calculated by subtracting LEAK respiration from ET capacity and then dividing the result by the ET capacity, as a measure of the degree of coupling (ATP generation).

### 4.4. ATP Quantification

ATP measurements were performed using a VICTOR Nivo multimode microplate reader (Perkin Elmer, Waltham, MA, USA) and the ATPlite Assay kit (Perkin Elmer, Waltham, MA, USA), according to the manufacturer’s instructions. Briefly, 15 μL lysed sample solution (corresponding to 50,000 mononuclear cells) was analyzed with 50 μL substrate solution and 35 μL PBS per well, in ½ area plates.

### 4.5. Data Analysis

Statistical analysis was performed using GraphPad PRISM software (GraphPad Software version 9.0). All data are expressed as mean ± SEM. To account for the presence of residual oxygen consumption all data were corrected for non-mitochondrial oxygen consumption [25] all statistical analyses (one-way or two-way ANOVA or mixed effects analysis with Bonferroni post hoc test) were performed on the antimycin-corrected data (*n* = 5 for the protocols using human platelets, *n* = 5–6 for the protocols using PMBCs and *n* = 3–4 for the protocols using HepG2 cells).

## 5. Conclusions

In human platelets and PBMCs and also in HepG2 cells, acute administration of amiodarone elicited a dose-dependent mitochondrial dysfunction through a dual mechanism both direct and through its metabolite DEA. By inhibiting maximum OXPHOS (mainly through complex II) and increasing the non-ATP-generating (LEAK) respiration, a cumulative decrease of P-L control efficiency occurred. Additionally, amiodarone elicited a time-dependent decrease of respiration in PBMCs and reduced ATP levels to a greater extent than did rotenone. As amiodarone is an effective antiarrhythmic, recommended by current guidelines [2,3], intracellular delivery of succinate may be a viable strategy to combat potential amiodarone-induced mitochondrial toxicity. Sotalol, another antiarrhythmic drug, did not elicit mitochondrial dysfunction in acute application.

## Data Availability

Data are contained within the article.

## Abbreviations

ADP	Adenosine diphosphate
ATP	Adenosine triphosphate
Cyt. c	Cytochrome c
CI	Complex I
CII	Complex II
DEA	Desethylamiodarone
DMSO	Dimethyl sulfoxide
ET	Electron transport
ETS	Electron transport system
FCCP	Carbonyl cyanide p-(trifluoromethoxy) phenylhydrazone
LEAK	Non-phosphorylating respiration
NADH	Nicotinamide adenine dinucleotide reduced form
OXPHOS	Oxidative phosphorylation
PBMCs	Peripheral blood mononuclear cells
ROS	Reactive oxygen species
ROX	Residual oxygen consumption

## References

[1] Fu D.G. (2015). Cardiac Arrhythmias: Diagnosis, Symptoms, and Treatments. Cell Biochem. Biophys..

[2] Soar J., Perkins G.D., Maconochie I., Böttiger B.W., Deakin C.D., Sandroni C., Olasveengen T.M., Wyllie J., Greif R., Lockey A. (2019). European Resuscitation Council Guidelines for Resuscitation: 2018 Update—Antiarrhythmic drugs for cardiac arrest. Resuscitation.

[3] Hindricks G., Potpara T., Dagres N., Arbelo E., Bax J.J., Blomstrom-Lundqvist C., Boriani G., Castella M., Dan G.A., Dilaveris P.E. (2020). 2020 ESC Guidelines for the diagnosis and management of atrial fibrillation developed in collaboration with the European Association of Cardio-Thoracic Surgery (EACTS). Eur. Heart J..

[4] Pallandi R.T., Campbell T.J. (1987). Resting, and rate-dependent depression of Vmax of guinea-pig ventricular action potentials by amiodarone and desethylamiodarone. Br. J. Pharmacol..

[5] Wu Q., Ning B., Xuan J., Ren Z., Guo L., Bryant M.S. (2016). The role of CYP 3A4 and 1A1 in amiodarone-induced hepatocellular toxicity. Toxicol. Lett..

[6] Adams P.C., Holt D.W., Storey G.C., Morley A.R., Callaghan J., Campbell R.W. (1985). Amiodarone and its desethyl metabolite: Tissue distribution and morphologic changes during long-term therapy. Circulation.

[7] Brien J.F., Jimmo S., Brennan F.J., Ford S.E., Armstrong P.W. (1987). Distribution of amiodarone and its metabolite, desethylamiodarone, in human tissues. Can. J. Physiol. Pharmacol..

[8] Kannan R., Sarma J.S., Guha M., Venkataraman K. (1989). Tissue drug accumulation and ultrastructural changes during amiodarone administration in rats. Fundam. Appl. Toxicol. Off. J. Soc. Toxicol..

[9] Basaria S., Cooper D.S. (2005). Amiodarone and the thyroid. Am. J. Med..

[10] Colby R., Geyer H. (2017). Amiodarone-induced pulmonary toxicity. JAAPA.

[11] Epstein A.E., Olshansky B., Naccarelli G.V., Kennedy J.I., Murphy E.J., Goldschlager N. (2016). Practical Management Guide for Clinicians Who Treat Patients with Amiodarone. Am. J. Med..

[12] Ramachandran A., Visschers R.G.J., Duan L., Akakpo J.Y., Jaeschke H. (2018). Mitochondrial dysfunction as a mechanism of drug-induced hepatotoxicity: Current understanding and future perspectives. J. Clin. Transl. Res..

[13] Silva Santos L.F., Stolfo A., Calloni C., Salvador M. (2017). Catechin and epicatechin reduce mitochondrial dysfunction and oxidative stress induced by amiodarone in human lung fibroblasts. J. Arrhythmia.

[14] Bolt M.W., Card J.W., Racz W.J., Brien J.F., Massey T.E. (2001). Disruption of mitochondrial function and cellular ATP levels by amiodarone and N-desethylamiodarone in initiation of amiodarone-induced pulmonary cytotoxicity. J. Pharmacol. Exp. Ther..

[15] Felser A., Blum K., Lindinger P.W., Bouitbir J., Krähenbühl S. (2013). Mechanisms of hepatocellular toxicity associated with dronedarone—A comparison to amiodarone. Toxicol. Sci..

[16] Fromenty B., Fisch C., Berson A., Letteron P., Larrey D., Pessayre D. (1990). Dual effect of amiodarone on mitochondrial respiration. Initial protonophoric uncoupling effect followed by inhibition of the respiratory chain at the levels of complex I and complex II. J. Pharmacol. Exp. Ther..

[17] Varbiro G., Toth A., Tapodi A., Veres B., Sumegi B., Gallyas F. (2003). Concentration dependent mitochondrial effect of amiodarone. Biochem. Pharm..

[18] Ehinger J.K., Piel S., Ford R., Karlsson M., Sjövall F., Frostner E., Morota S., Taylor R.W., Turnbull D.M., Cornell C. (2016). Cell-permeable succinate prodrugs bypass mitochondrial complex I deficiency. Nat. Commun..

[19] Piel S., Ehinger J.K., Chamkha I., Frostner E., Sjövall F., Elmér E., Hansson M.J. (2018). Bioenergetic bypass using cell-permeable succinate, but not methylene blue, attenuates metformin-induced lactate production. Intensive Care Med. Exp..

[20] Avram V.F., Chamkha I., Åsander-Frostner E., Ehinger J.K., Timar R.Z., Hansson M.J., Muntean D.M., Elmér E. (2021). Cell-Permeable Succinate Rescues Mitochondrial Respiration in Cellular Models of Statin Toxicity. Int. J. Mol. Sci..

[21] Owiredu S., Ranganathan A., Eckmann D.M., Shofer F.S., Hardy K., Lambert D.S., Kelly M., Jang D.H. (2020). Ex vivo use of cell-permeable succinate prodrug attenuates mitochondrial dysfunction in blood cells obtained from carbon monoxide-poisoned individuals. Am. J. Physiol. Cell Physiol..

[22] Kerin N.Z. (2018). Intravenous Sotalol: An Under Used Treatment Strategy. Cardiology.

[23] Petrus A.T., Lighezan D.L., Danila M.D., Duicu O.M., Sturza A., Muntean D.M., Ionita I. (2019). Assessment of platelet respiration as emerging biomarker of disease. Physiol. Res..

[24] Sjövall F., Ehinger J.K., Marelsson S.E., Morota S., Frostner E.A., Uchino H., Lundgren J., Arnbjörnsson E., Hansson M.J., Fellman V. (2013). Mitochondrial respiration in human viable platelets--methodology and influence of gender, age and storage. Mitochondrion.

[25] Gnaiger E. (2020). Mitochondrial Pathways and Respiratory Control an Introduction to OXPHOS Analysis. Bioenerg. Commun..

[26] Wallace K.B. (2015). Multiple Targets for Drug-Induced Mitochondrial Toxicity. Curr. Med. Chem..

[27] Fromenty B., Fisch C., Labbe G., Degott C., Deschamps D., Berson A., Letteron P., Pessayre D. (1990). Amiodarone inhibits the mitochondrial beta-oxidation of fatty acids and produces microvesicular steatosis of the liver in mice. J. Pharmacol. Exp. Ther..

[28] Kaufmann P., Török M., Hänni A., Roberts P., Gasser R., Krähenbühl S. (2005). Mechanisms of benzarone and benzbromarone-induced hepatic toxicity. Hepatology.

[29] Spaniol M., Bracher R., Ha H.R., Follath F., Krähenbühl S. (2001). Toxicity of amiodarone and amiodarone analogues on isolated rat liver mitochondria. J. Hepatol..

[30] Serviddio G., Bellanti F., Giudetti A.M., Gnoni G.V., Capitanio N., Tamborra R., Romano A.D., Quinto M., Blonda M., Vendemiale G. (2011). Mitochondrial oxidative stress and respiratory chain dysfunction account for liver toxicity during amiodarone but not dronedarone administration. Free Radic. Biol. Med..

[31] Kushnareva Y., Newmeyer D.D. (2010). Bioenergetics and cell death. Ann. N. Y. Acad. Sci..

[32] Waldhauser K.M., Török M., Ha H.R., Thomet U., Konrad D., Brecht K., Follath F., Krähenbühl S. (2006). Hepatocellular toxicity and pharmacological effect of amiodarone and amiodarone derivatives. J. Pharmacol. Exp. Ther..

[33] Stepanova A., Shurubor Y., Valsecchi F., Manfredi G., Galkin A. (2016). Differential susceptibility of mitochondrial complex II to inhibition by oxaloacetate in brain and heart. Biochim. Biophys. Acta.

[34] Risiglione P., Leggio L., Cubisino S.A.M., Reina S., Paternò G., Marchetti B., Magrì A., Iraci N., Messina A. (2020). High-Resolution Respirometry Reveals MPP(+) Mitochondrial Toxicity Mechanism in a Cellular Model of Parkinson’s Disease. Int. J. Mol. Sci..

[35] Karkhanis A., Leow J.W.H., Hagen T., Chan E.C.Y. (2018). Dronedarone-Induced Cardiac Mitochondrial Dysfunction and Its Mitigation by Epoxyeicosatrienoic Acids. Toxicol. Sci..

[36] Takai S., Oda S., Tsuneyama K., Fukami T., Nakajima M., Yokoi T. (2016). Establishment of a mouse model for amiodarone-induced liver injury and analyses of its hepatotoxic mechanism. J. Appl. Toxicol. JAT.

[37] Guo L., Rondina M.T. (2019). The Era of Thromboinflammation: Platelets Are Dynamic Sensors and Effector Cells During Infectious Diseases. Front. Immunol..

[38] Sjövall F., Morota S., Persson J., Hansson M.J., Elmér E. (2013). Patients with sepsis exhibit increased mitochondrial respiratory capacity in peripheral blood immune cells. Crit Care.

[39] Fromenty B., Letteron P., Fisch C., Berson A., Deschamps D., Pessayre D. (1993). Evaluation of human blood lymphocytes as a model to study the effects of drugs on human mitochondria. Effects of low concentrations of amiodarone on fatty acid oxidation, ATP levels and cell survival. Biochem. Pharm..

[40] Lafuente-Lafuente C., Alvarez J.C., Leenhardt A., Mouly S., Extramiana F., Caulin C., Funck-Brentano C., Bergmann J.F. (2009). Amiodarone concentrations in plasma and fat tissue during chronic treatment and related toxicity. Br. J. Clin. Pharmacol..

[41] Zahno A., Brecht K., Morand R., Maseneni S., Török M., Lindinger P.W., Krähenbühl S. (2011). The role of CYP3A4 in amiodarone-associated toxicity on HepG2 cells. Biochem. Pharm..

[42] Lynch T., Price A. (2007). The effect of cytochrome P450 metabolism on drug response, interactions, and adverse effects. Am. Fam. Physician.

[43] Ogu C.C., Maxa J.L. (2000). Drug interactions due to cytochrome P450. Baylor University Medical Center Proceedings.

[44] Novotna A., Dvorak Z. (2014). Omeprazole and lansoprazole enantiomers induce CYP3A4 in human hepatocytes and cell lines via glucocorticoid receptor and pregnane X receptor axis. PLoS ONE.

[45] Pascussi J.-M., Drocourt L., Gerbal-Chaloin S., Fabre J.-M., Maurel P., Vilarem M.-J. (2001). Dual effect of dexamethasone on CYP3A4 gene expression in human hepatocytes. Eur. J. Biochem..

[46] Chistiakov D.A., Sobenin I.A., Revin V.V., Orekhov A.N., Bobryshev Y.V. (2014). Mitochondrial aging and age-related dysfunction of mitochondria. BioMed. Res. Int..

[47] Dai D.-F., Chen T., Johnson S.C., Szeto H., Rabinovitch P.S. (2012). Cardiac aging: From molecular mechanisms to significance in human health and disease. Antioxid Redox Signal.

[48] Muscari C., Caldarera C.M., Guarnieri C. (1990). Age-dependent production of mitochondrial hydrogen peroxide, lipid peroxides and fluorescent pigments in the rat heart. Basic Res. Cardiol..

[49] Saadeh K., Fazmin I.T. (2021). Mitochondrial Dysfunction Increases Arrhythmic Triggers and Substrates; Potential Anti-arrhythmic Pharmacological Targets. Front. Cardiovasc. Med..

[50] Kashani A., Phillips C.O., Foody J.M., Wang Y., Mangalmurti S., Ko D.T., Krumholz H.M. (2006). Risks Associated With Statin Therapy. Circulation.

[51] Hirota T., Ieiri I. (2015). Drug-drug interactions that interfere with statin metabolism. Expert Opin. Drug Metab. Toxicol..

[52] Bouitbir J., Sanvee G.M., Panajatovic M.V., Singh F., Krahenbuhl S. (2019). Mechanisms of statin-associated skeletal muscle-associated symptoms. Pharmacol. Res..

[53] Apostolopoulou M., Corsini A., Roden M. (2015). The role of mitochondria in statin-induced myopathy. Eur. J. Clin. Investig..

[54] Durhuus J.A., Hansson S., Morville T., Kuhlman A.B., Dohlmann T.L., Larsen S., Helge J.W., Angleys M., Muniesa-Vargas A., Bundgaard J.R. (2020). Simvastatin improves mitochondrial respiration in peripheral blood cells. Sci. Rep..

[55] Piel S., Chamkha I., Dehlin A.K., Ehinger J.K., Sjövall F., Elmér E., Hansson M.J. (2020). Cell-permeable succinate prodrugs rescue mitochondrial respiration in cellular models of acute acetaminophen overdose. PLoS ONE.

[56] Gnaiger E., Kuznetsov A.V., Schneeberger S., Seiler R., Brandacher G., Steurer W., Margreiter R. Mitochondria in the Cold. Proceedings of Life in the Cold.

[57] Avram V.F., Bîna A.M., Sima A., Aburel O.M., Sturza A., Burlacu O., Timar R.Z., Muntean D.M., Elmér E., Crețu O.M. (2021). Improvement of Platelet Respiration by Cell-Permeable Succinate in Diabetic Patients Treated with Statins. Life.

